# Supplementary material for: The Allosteric Communication Network in the Activation of Antithrombin by Heparin
Source: Int J Mol Sci. 2025 Sep 15;26(18):8984. doi: 10.3390/ijms26188984 (PMC12469680; doi:10.3390/ijms26188984)
Supplement: Supplementary file 1 [file ijms-26-08984-s001.zip › ijms-3670133-supplementary.pdf]

International Journal of
Molecular Sciences

MDPI

Supplement

The Allosteric Communication Network in the Activation of Antithrombin by Heparin

Gonzalo Izaguirre [1,2]

[1] Insight-DNA, Oak Park, IL 60302, USA; izaguirre.g@insight-dna.net
[2] College of Dentistry, University of Illinois Chicago, Chicago, IL 60612, USA

S1. Introduction

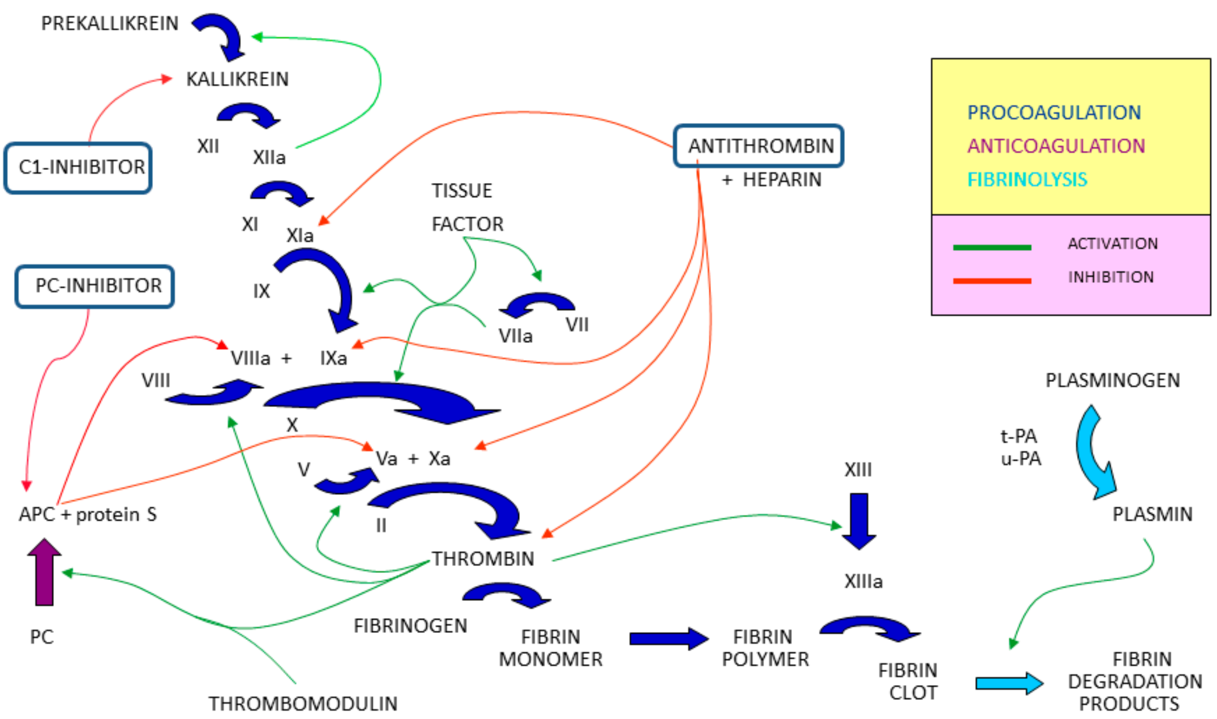

Figure S1. Antithrombin is the main regulator of the blood coagulation cascade. The AT-heparin complex inhibits several procoagulant proteases. Other serpins (C1-inhibitor, and PC-inhibitor) are also regulators of the coagulation cascade by inhibiting pro- or anticoagulation proteases. Additional serpins that also regulate the coagulation cascade include heparin cofactor II (HCII), plasminogen activator inhibitors 1 and 2 (PAI-1 and PAI-2), protein nexin 1 (PN-1), and protein Z-dependent inhibitor (ZPI).

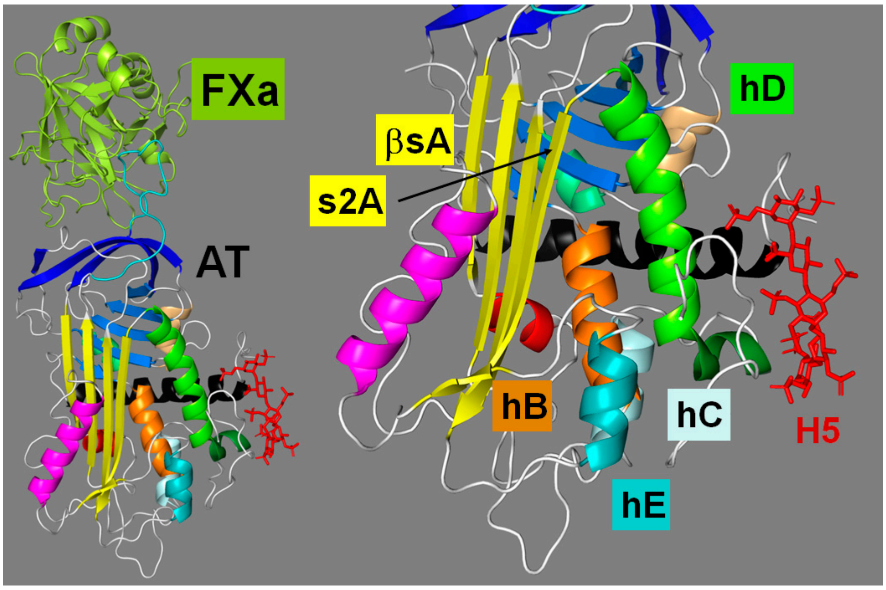

Figure S2. Antithrombin structure. The Michaelis complex that is formed by antithrombin (AT), heparin pentasaccharide (H5), and factor Xa is shown on the left panel (PDB code 2GD4 [2]). The right panel shows a close-up of the AT structure depicting the heparin binding site and other regions involved in the allosteric communication network.

S2. Results

*S2.1. Identification of a network of residues that undergo the largest positional changes during the R-to-AH transition***.**

Table S1. **Comparative structural analysis of the HBS between the heparin-free and H5-bound antithrombin forms**. Crystal structures of the AT R (PDB code 1T1F) and AH (PDB code 1EO3) forms were aligned by overlaying only the backbone atoms down to an RMS of 1.48 Ȧ. The distance of the most distal carbon atom for each residue side chain between the two structures was measured. In blue are depicted the positively charged residues that contact the negatively charged heparin. The largest distances are displayed in red. Helix P is the extension of helix D that occurs in the AH form. Positively charged residues in this section bind longer heparin chains.

Pentasaccharide binding residues			N-tail		helix D		L126	1.1 Å
K11	5.6 Å	N-tail	C8	2.2 Å	K114	5.6 Å	N127	2.4 Å
R13	3.5 Å		T9	2.9 Å	T115	2.6 Å	C128	3.2 Å
R46	9.8 Å	helix A	A10	3.3 Å	S116	3.3 Å	R129	5.5 Å
K114	5.6 Å	hC-hD loop	K11	5.6 Å	D117	2.8 Å	L130	2.2 Å
F121	4.2 Å	helix D	P12	3.8 Å	Q118	2.6 Å	helix P	
F122	3.2 Å		R13	3.5 Å	I119	1.5 Å	Y131	**7.0 Å**
K125	3.5 Å		D14	3.1 Å	H120	**4.9 Å**	R132	3.1 Å
R129	5.5 Å		I15	3.0 Å	F121	4.2 Å	K133	**15.9 Å**
			P16	1.3 Å	F122	3.2 Å	A134	**8.4 Å**
			M17	**4.0 Å**	F123	2.0 Å	N135	3.2 Å
			N18	3.5 Å	A124	1.8 Å	K136	**7.7 Å**
			P19	1.0 Å	K125	3.5 Å	S137	1.9 Å
			M20	0.8 Å				
			C21	0.9 Å				

Table S2. Comparative structural analysis of the protein core between the heparin-free and H5-bound antithrombin forms. Crystal structures of the AT R (PDB code 1T1F) and AH (PDB code 1EO3) forms were aligned by overlaying only the backbone atoms down to an RMS of 1.48 Å. The distance of the most distal carbon atom for each residue side chain between the two structures was measured. The largest distances are displayed in red.

helix B		strand 2A		helix E		strand 1A		strand 3A	
P80	1.0 Å	S138	1.8 Å	F154	1.0 Å	G167	-- Å	L213	0.7 Å
L81	1.9 Å	K139	**3.9 Å**	N155	0.5 Å	A168	2.6 Å	V214	1.2 Å
S82	1.8 Å	L140	1.9 Å	E156	0.6 Å	K169	2.3 Å	L215	1.2 Å
I83	0.8 Å	V141	3.3 Å	T157	1.1 Å	L170	0.7 Å	V216	0.3 Å
S84	0.6 Å	S142	**3.4 Å**	Y158	0.7 Å	Q171	0.9 Å	N217	0.7 Å
T85	0.7 Å	A143	2.2 Å	Q159	0.7 Å	P172	0.8 Å	T218	1.1 Å
A86	0.8 Å	N144	**3.7 Å**	D160	1.4 Å			I219	1.6 Å
F87	0.6 Å			I161	1.0 Å			Y220	**3.6 Å**
A88	0.8 Å			S162	1.1 Å			F221	1.3 Å
M89	**5.0 Å**			E163	2.3 Å			K222	**5.6 Å**
T90	1.0 Å			L164	1.9 Å			G223	-- Å
K91	1.8 Å			loop				L224	1.5 Å
L92	1.0 Å			V165	1.3 Å			W225	1.0 Å
G93	-- Å			Y166	**6.6 Å**			K226	1.4 Å
A94	0.8 Å								
C95	1.6 Å								

Table S3. Comparative structural analysis of relevant residue groups between the heparin-free and H5-bound antithrombin forms. Crystal structures of the AT R (PDB code 1T1F) and AH (PDB code 1EO3) forms were aligned by overlaying only the backbone atoms down to an RMS of 1.48 Å. The distance of the most distal carbon atom for each residue side chain between the two structures was measured. The largest distances are displayed in red.

Y131 pocket		S380 pocket		E381 network	
Y131	7.0 Å	S380	9.4 Å	E381	6.7 Å
L81	1.7 Å	F221	1.3 Å	K139	3.9 Å
L130	2.1 Å	F274	1.1 Å	K222	5.6 Å
N127	2.4 Å	L373	0.7 Å	E374	3.0 Å
S142	3.4 Å	V375	0.3 Å	Y220	3.6 Å
I219	2.1 Å			F372	3.7 Å
T85	0.7 Å			E195	2.7 Å
L140	1.9 Å			R197	6.3 Å

S2.2. Mutations in the identified residues increased native antithrombin reactivity with factor Xa.

Table S4. **Inhibition of thrombin by antithrombin variants**. Second order association rate constants and stoichiometry of inhibition values were determined as described in the Materials and Methods section.

	Second Order Rate Constants (M^{-1} s^{-1})	Fold change	Stoichiometry of Inhibition
**Wild Type**	(1.1 ± 0.1) x 10^4	1	1.2 ± 0.1
**M17V**	(5.6 ± 0.9) x 10^3	0.51	0.97 ± 0.07
**M89V**	(6.5 ± 0.9) x 10^3	0.59	9.5 ± 0.1
**H120V**	(7.4 ± 1.4) x 10^3	0.67	6.3 ± 0.4
**N144V**	(7.5 ± 1.3) x 10^3	0.68	1.2 ± 0.1
**Y166V**	(7.6 ± 1.0) x 10^3	0.69	62 ± 3
**Y131L**	ND		
**H120L - Y131L**	(8.1 ± 0.9) x 10^3	0.74	48 ± 3
**H120 L - Y166L**	(3.8 ± 0.4) x 10^3	0.35	7.2 ± 0.3
**Y131L - Y166L**	(6.8 ± 2.0) x 10^3	0.62	6.0 ± 1
**H120L - Y131L - Y166L**	(5.0 ± 0.6) x 10^3	0.45	55 ± 3
**E381G**	ND		
**E195R - R197E (Switch)**	(3.6 ± 1.2) x 10^3	0.33	1.3 ± 0.2
**Switch - S380G**	ND		
**Y131L - S380G**	ND		

Table S5. Inhibition of factor Xa by antithrombin variants. Second order association rate constants and stoichiometry of inhibition values were determined as described in the Materials and Methods section.

	Second Order Rate Constants (M^{-1} s^{-1})	Fold change	Stoichiometry of Inhibition
**Wild Type**	(3.9 ± 0.8) x 10^3	1	1.2 ± 0.2
**M17V**	(5.3 ± 0.2) x 10^3	1.4	1.2 ± 0.0
**M89V**	(3.7 ± 0.5) x 10^4	9.5	15 ± 2
**H120V**	(9.2 ± 1.2) x 10^5	236	12 ± 1
**N144V**	(2.3 ± 0.1) x 10^3	0.59	1.5 ± 0.0
**Y166V**	(2.5 ± 0.8) x 10^5	64	84 ± 2
**Y131L**	(1.2 ± 0.1) x 10^5	31	6.7 ± 0.4
**H120L - Y131L**	(2.5 ± 0.8) x 10^5	64	60 ± 6
**H120 L - Y166L**	(2.7 ± 0.3) x 10^5	69	15 ± 1
**Y131L - Y166L**	(7.4 ± 1.3) x 10^4	19	7.1 ± 1
**H120L - Y131L - Y166L**	(2.1 ± 0.6) x 10^5	54	58 ± 5
**E381G**	(1.0 ± 0.1) x 10^3	0.26	4.6 ± 0.5
**E195R - R197E (Switch)**	(1.4 ± 0.7) x 10^4	3.6	1.8 ± 0.4
**Switch - S380G**	(2.8 ± 1.3) x 10^5	72	4.5 ± 0.1
**Y131L - S380G**	(3.7 ± 0.3) x 10^5	95	3.2 ± 0.1

Table S6. Inhibition of factor Xa by antithrombin variants with heparin pentasaccharide. Second order association rate constants and stoichiometry of inhibition values were determined as described in the Materials and Methods section.

	Second Order Rate Constants (M^{-1} s^{-1})	Fold change	Stoichiometry of Inhibition
**Wild Type**	(1.4 ± 0.3) x 10^6	1	1.4 ± 0.3
**M17V**	(7.9 ± 0.6) x 10^5	0.56	1.7 ± 0.0
**M89V**	(1.2 ± 0.3) x 10^6	0.86	23 ± 4
**H120V**	(2.4 ± 0.2) x 10^6	1.7	13 ± 0
**N144V**	(3.2 ± 0.2) x 10^5	0.23	1.9 ± 0.1
**Y166V**	(1.3 ± 0.3) x 10^6	0.93	120 ± 20
**Y131L**	(1.5 ± 0.2) x 10^6	1.1	4.7 ± 0.3
**H120L - Y131L**	(7.7 ± 0.7) x 10^5	0.55	44 ± 1
**H120 L - Y166L**	(8.8 ± 2.4) x 10^5	0.63	16 ± 3
**Y131L - Y166L**	(1.6 ± 0.3) x 10^6	1.1	7.1 ± 0.2
**H120L - Y131L - Y166L**	(5.6 ± 1.6) x 10^5	0.40	54 ± 2
**E381G**	(1.4 ± 0.1) x 10^5	0.10	5.9 ± 0.1
**E195R - R197E (Switch)**	(1.6 ± 0.2) x 10^6	1.1	3.3 ± 0.3
**Switch - S380G**	(1.0 ± 0.2) x 10^6	0.71	5.7 ± 0.4
**Y131L - S380G**	(1.9 ± 0.3) x 10^6	1.4	3.5 ± 0.1

S2.3. Functional cooperation among ACN residues.

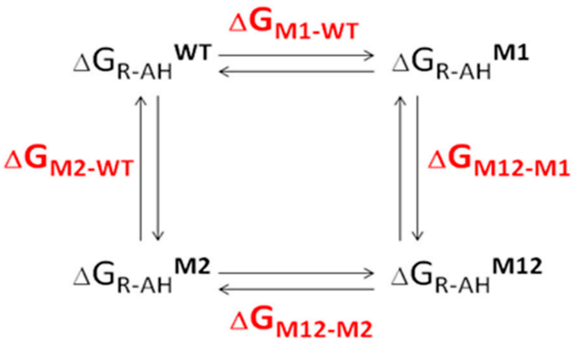

$$\Delta G_{M\text{-}WT} = \Delta G_{R\text{-}AH}{}^{M} - \Delta G_{R\text{-}AH}{}^{WT} = -RT\ \mathrm{Ln}\ \frac{K^{*}_{M}}{K^{*}_{WT}}$$

$$\Delta G_{12} = -RT\ \mathrm{Ln}\ \frac{K^{*}_{M12}\,K^{*}_{WT}}{K^{*}_{M1}\,K^{*}_{M2}}$$

Figure S3. Double mutant thermodynamic cycle for antithrombin activation. Free energy of activation is represented by the free energy difference between the R and AH antithrombin forms and calculated from the difference in their second order association rate constant values for the inhibition of factor Xa. The differences in free energy of activation between the wildtype (WT), single mutants (M1, M2), and the double mutant (M12) represent the effect of the mutations on activation.

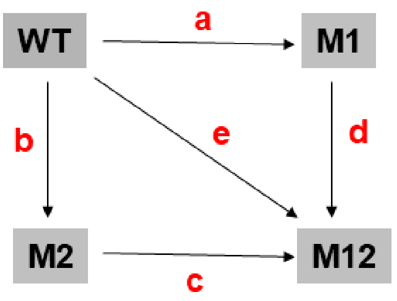

$$\text{Coupling index} = \frac{c}{a} = \frac{d}{b}$$

Figure S4. Site-specific double-mutant thermodynamic coupling analysis scheme. Mutants 1 and 2 can be any number of combined mutations where *a-e* are ratios of the second order association rate constant values for inhibition of factor Xa. Coupling index (CI) values equal to *1* denote no thermodynamic linkage between mutations 1 and 2. CI values larger than *1* indicate the functional cooperative linkage between the residues.

Table S7. Thermodynamic coupling of ACN residues. The effect of combined mutations on antithrombin reactivity with factor Xa was tested for cooperative or additive effects between the residues in green versus those in purple. A coupling index value of 1 indicates additivity and a value above 1 indicates cooperative linkage.

M1 vs M2	a	b	c	d	e	Coupling Index
H120V Y131L	230	30	2.1	0.27	63	110
H120V Y166V	230	63	1	0.27	63	230
Y131L Y166V	30	63	0.29	0.62	18	100
H120L - Y131L Y166V	63	63	0.84	0.84	53	1
H120L - Y166L Y131L	68	30	1.8	0.78	53	38
Y131L - Y166L H120V	18	230	0.23	2.8	53	81

S2.4. Protein physical property changes associated with the R-to-A transitions.*

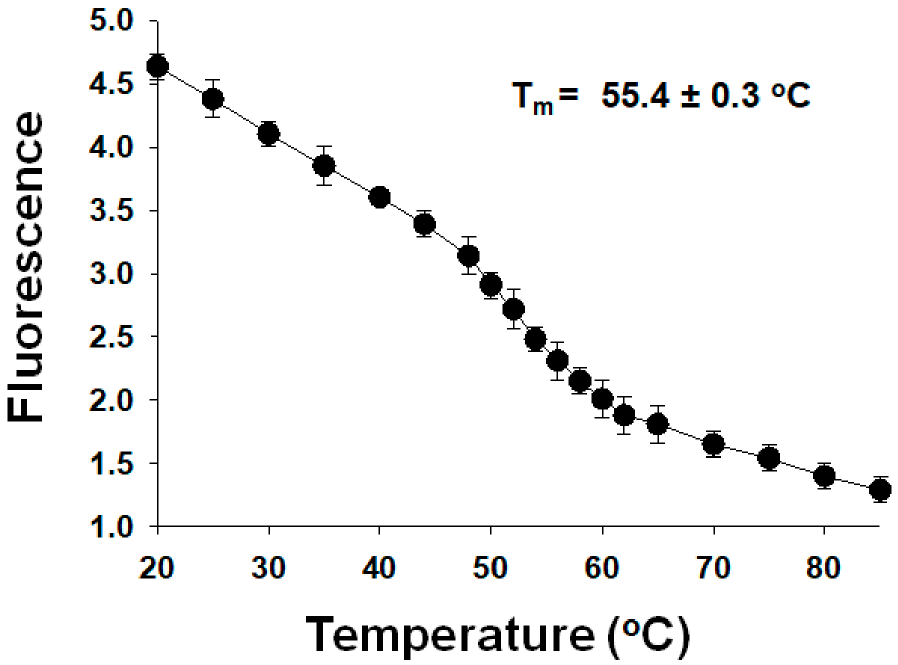

Figure S5. Thermal denaturation of the antithrombin protein. Denaturation of AT variants was monitored by changes in tryptophan fluorescence as a dependence of increments in temperature. Fluorescence was measured at wavelengths: excitation = 280 nm, emission = 345 nm under conditions described in the Materials and Methods section. This plot corresponds to the average values of two experiments performed with the mutant Y131L-Y166L and is accompanied by the standard deviation values.

Table S8. Physical properties of the antithrombin ACN mutation variants. Protein melting temperatures (T_m) were determined from the dependence of the tryptophane fluorescence (excitation λ=280, emission λ=345) on changes in temperature. Dependence curves were fitted to the van't Hoff equation to extract T_m values. Protein baseline fluorescence was determined as the slope of the linear dependence of fluorescence changes on the antithrombin concentration (FU/nM) and is also shown as the percentage relative to the value of the wild-type protein. The gain in tryptophane fluorescence upon heparin binding was determined by adding a saturating concentration of high affinity heparin pentasaccharide that was five-fold larger than the protein concentration. The fluorescence gain is shown as the percentage relative to the native baseline fluorescence and as the percentage when compared to the fluorescence change observed with the wild-type protein. The conditions in which these experiments were conducted are described in the Materials and Methods section. Experimental values correspond to the average of at least two experiments and are accompanied by the associated standard deviation values.

AT variant	Melting Temperature T_m (°C)	Degree Change (°C)	Baseline Fluorescence (FU/nM) (%)	Fluorescence Gain FU % (% WT)
**Wild Type**	56.4 ± 0.0	0	75 ± 1 (100)	42 (100)
**M17V**	58.2 ± 0.1	1.80	80 ± 3 (107)	39 (93)
**M89V**	58.8 ± 0.0	2.40	36 ± 1 (48)	1.8 (4.3)
**H120V**	49.1 ± 1.1	-7.30	140 ± 6 (187)	1.7 (4.0)
**N144V**	61.0 ± 0.0	4.60	78 ± 3 (104)	17 (40)
**Y166V**	52.0 ± 1.2	-4.40	80 ± 1 (107)	0 (0)
**Y131L**	56.2 ± 0.8	-0.20	78 ± 1 (104)	5.8 (14)
**H120L – Y131L**	57.0 ± 0.8	0.60	34 ± 1 (45)	0 (0)
**H120L – Y166L**	54.8 ± 1.0	-1.60	63 ± 2 (84)	0 (0)
**Y131L – Y166L**	55.4 ± 0.3	-1.00	47 ± 1 (63)	11 (26)
**H120L – Y131L – Y166**	44.1 ± 1.1	-12.3	58 ± 3 (77)	0 (0)

S2.6. Molecular dynamic simulation of the antithrombin ACN mutants.

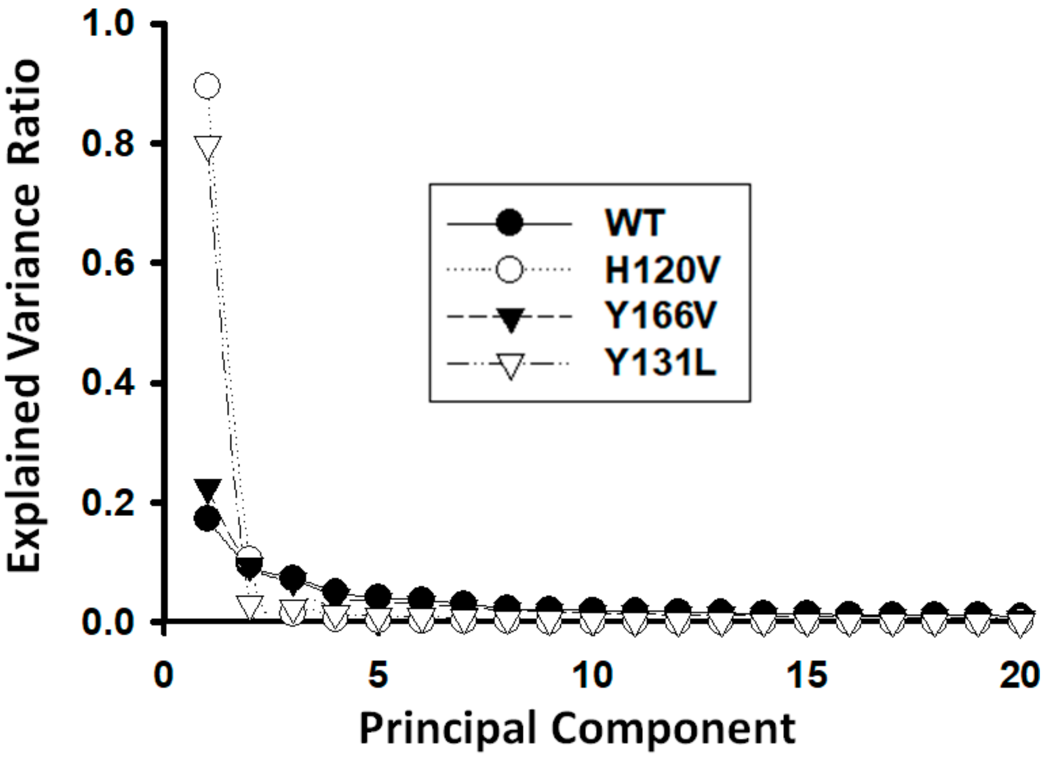

Figure S6. Variance distribution of principal component analysis (PCA) of Cα atom dynamics for wild-type (WT) and mutant simulations. The plots show the fraction of total variance explained by the first 20 principal components (PCs) derived from PCA of the backbone Cα atoms for the WT and mutant simulations. For the H120V, and Y131L simulations, PC1 alone explains over 90 and 80 %, respectively, of the total variance, indicating that the conformational dynamics are largely constrained to a single dominant mode of motion. In contrast, for the WT and Y166V simulations, variance is more spread across the PCs, suggesting a broader distribution of motion across multiple independent modes. These results highlight a fundamental shift in conformational flexibility and collective dynamics induced by the H120V mutation.

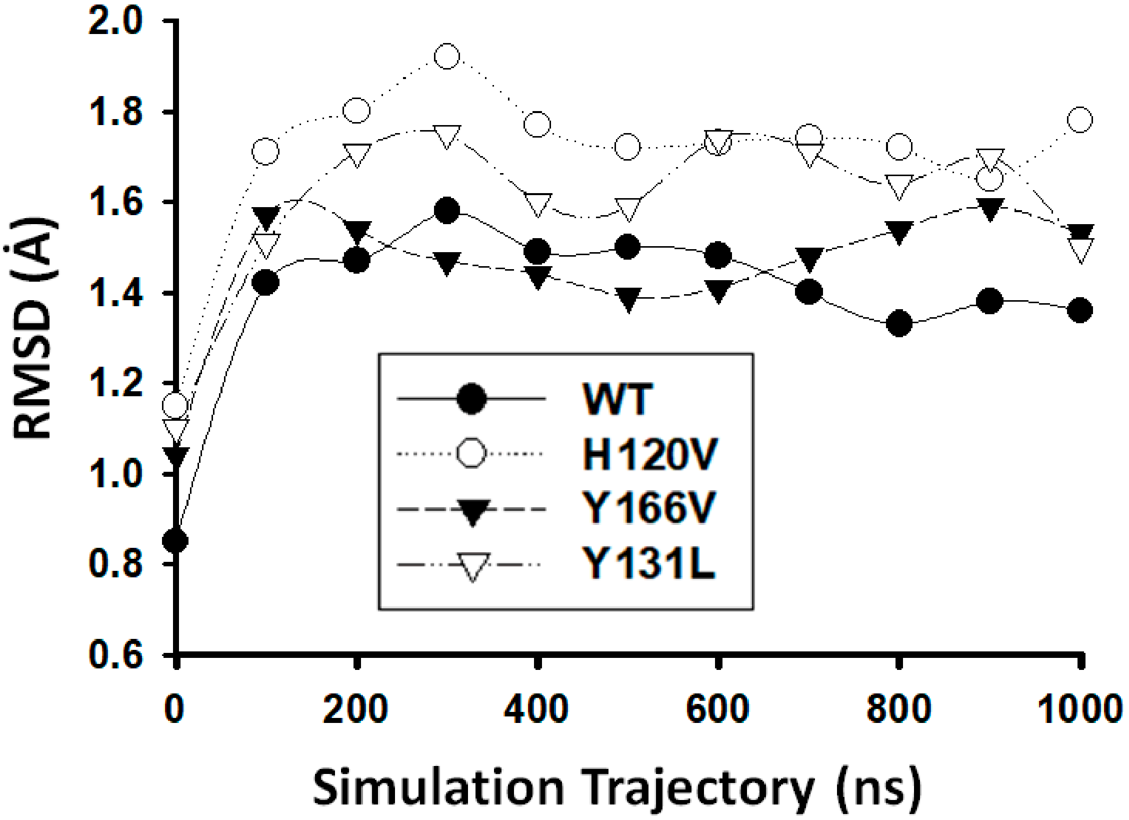

Figure S7. Comparison of RMSD evolution over time between WT and mutant antithrombin simulations. The plot shows the root mean square deviation (RMSD) in Ångströms (Å) for wild-type (WT) and mutant antithrombin structures during molecular dynamics simulations from 0 to 1000 ns. RMSD values at 0 ns correspond to the differences between the pre- and minimized structures. All other values reflect the difference between the minimized and simulated structures, thereby representing structural drift over time. The WT simulation shows the lowest initial and further drift values contrasting with the H120V simulation that shows the largest values.

Table S9. **Top positive and negative Pearson correlation differences between residue pairs in antithrombin wild-type and H120V molecular dynamics simulations.** This table lists the residue pairs that exhibit the largest changes in Pearson correlation coefficients between the wild-type (AT_WT) and mutant (H120V) antithrombin molecular dynamics (MD) simulations. Each row corresponds to a residue-residue pair identified by their residue indices (Residue_i and Residue_j), along with a ΔPearson value representing the difference in dynamic correlation strength between

the two simulations and the secondary structure location of each residue. MD Simulation Setup: All-atom molecular dynamics simulations of AT_WT and H120V antithrombin were performed in explicit solvent using OpenMM with the AMBER14 force field and a Langevin integrator. Simulations were conducted at 310 K for 1000 ns with a 2 fs time step, and conformational snapshots were saved every 20 ps. Correlation Matrix Calculation: The backbone Cα atom positions were extracted from the trajectories using MDTraj. Pearson correlation coefficients were computed pairwise for all Cα atoms over the full trajectory, resulting in a symmetric correlation matrix per simulation. ΔPearson Calculation: The difference matrix was generated by subtracting the AT_WT Pearson correlation matrix from the H120V matrix. Positive values indicate increased dynamic correlation in H120V relative to WT, while negative values represent decreased correlation. Table Content: The top-ranking residue pairs with the highest positive and negative ΔPearson values are reported. Each entry is sorted by absolute ΔPearson magnitude. These results highlight specific regions in the protein where dynamic coupling is significantly altered by the H120V mutation, offering insight into potential allosteric or structural impacts on antithrombin function.

ΔPearson	Residue _ i	Residue _ j	Secundary structure _ i	Secundary structure _ j
1.208897	397	395	RCL	RCL
1.203892	339	184	hI	hF
1.156366	396	395	RCL	RCL
1.150069	184	334	hF	hI
1.145333	183	339	hF	hI
1.125157	187	339	hF	hI
1.117962	339	188	hI	hF
1.116487	180	339	hF	hI
1.09781	334	183	hI	hF
1.093925	187	334	hF	hI
1.093593	212	371	s3A	s5A
1.087189	325	361	s6A	hI - s5A
1.080921	196	359	hF - s3A	hI - s5A
1.072902	361	196	hI - s5A	hF - s3A
1.067375	188	334	hF	hI
1.065279	196	360	hF - s3A	hI - s5A
1.062379	17	357	N- tail	hI
1.053525	325	362	s6A	hI - s5A
1.050037	360	325	hI - s5A	s6A
1.048895	184	338	hF	hI
1.048236	17	358	N - tail	hI - s5A
1.047658	180	334	hF	hI
1.047658	334	180	hI	hF
1.039612	187	333	hF	hI
1.038249	212	216	s3A	s3A
1.037499	198	339	hF - s3A	hI

ΔPearson	Residue _ i	Residue _ j	Secondary structure _ i	Secondary structure _ j
-1.471012	193	196	hF	hF- s3A
-1.390505	193	188	hF	hF
-1.351454	188	192	hF	hF
-1.322649	193	197	hF	hF -s3A
-1.302809	196	192	hF - s3A	hF
-1.291874	63	58	hA	hA
-1.289537	63	61	hA	hA
-1.285559	63	57	hA	hA
-1.276811	187	193	hF	hF
-1.269596	203	180	hF - s3A	hF
-1.259006	202	184	hF - s3A	hF
-1.22489	192	187	hF	hF
1.208897	395	397	RCL	RCL
-1.206322	202	183	hF - s3A	hF
-1.206045	192	197	hF	hF -s3A
1.203892	339	184	hI	hF
-1.201023	361	148	hI - s5A	s2A
-1.20028	193	189	hF	hF
-1.184741	190	193	hF	hF
-1.174393	178	184	s1A	hF
-1.169156	361	212	hI - s5A	s3A
-1.167203	361	147	hI - s5A	s2A
-1.165056	203	184	hF - s3A	hF
-1.15917	184	172	hF	s1A

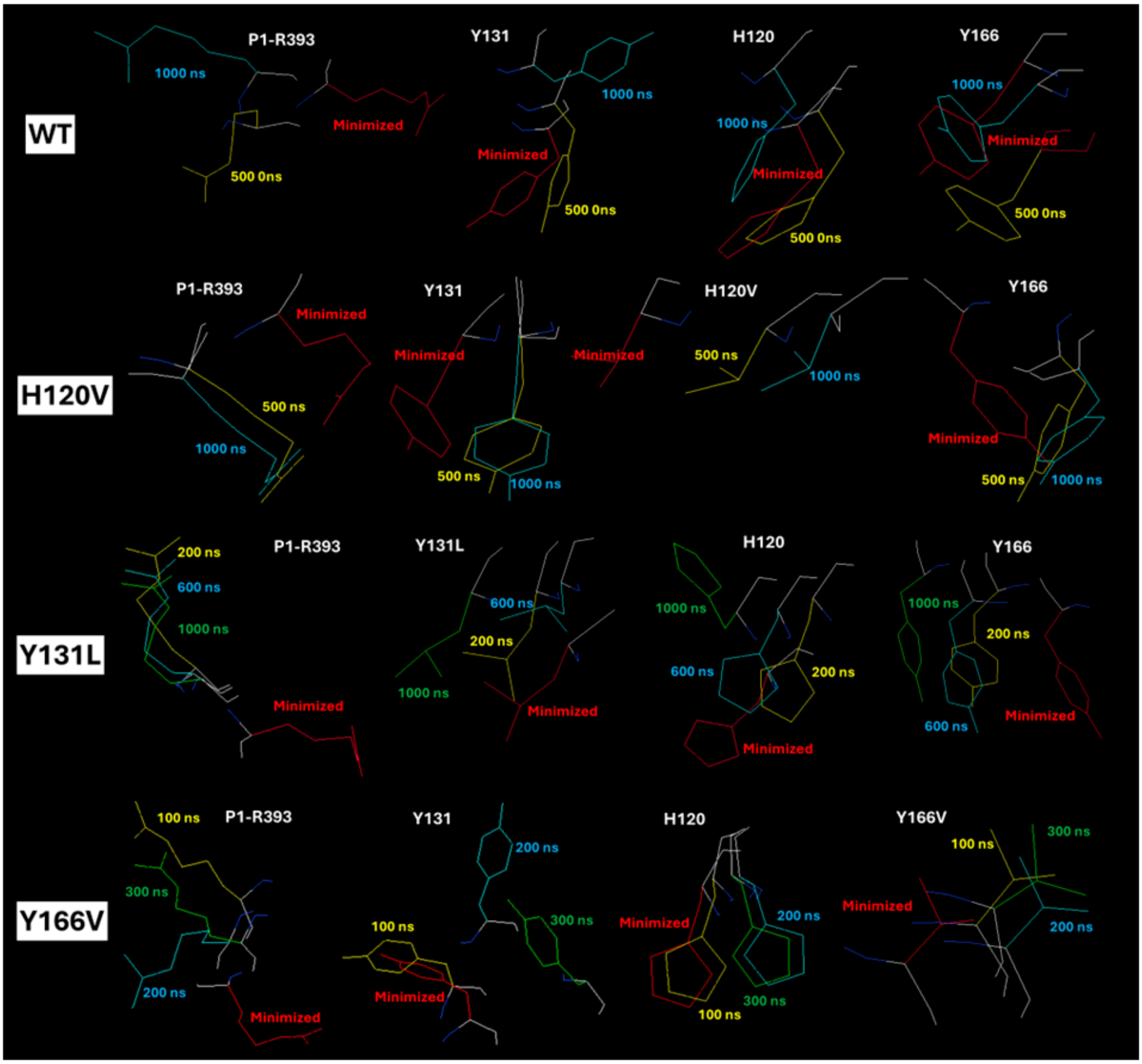

Figure S8. Structural evolution of key residues in wild-type (WT), H120V, Y131L, and Y166V antithrombin (AT) molecular dynamics simulations (MDS). Superimposed representations of selected residues from the WT, H120V, Y131L, and Y166V AT structures illustrate conformational changes during MDS. Structures are shown at key timepoints across the energy minimized (0 ns) to 1000 ns trajectories. Panels include the reactive center loop residue P1-R393, as well as H120, Y131, and Y166 or their mutant forms, which comprise the evolutionarily conserved allosteric communication network (ACN). All structures were aligned based on the backbone scaffold. Structural deviations from the energy minimized state (red) reflect progressive conformational adaptation unique to each mutant background. Notably, in the WT set, the P1-R393 and Y131 residues show longer motion compared to H120 and Y166, which remain more static. On

the contrary, in the H120V set, H120V and Y166 show longer motion than the other two residues. In the Y131L set, H120 and Y166 show progressive motion over the whole trajectory. In the Y166V set, P1-R393 and Y131 show the largest motion occurring early in the trajectory.

S3. Discussion

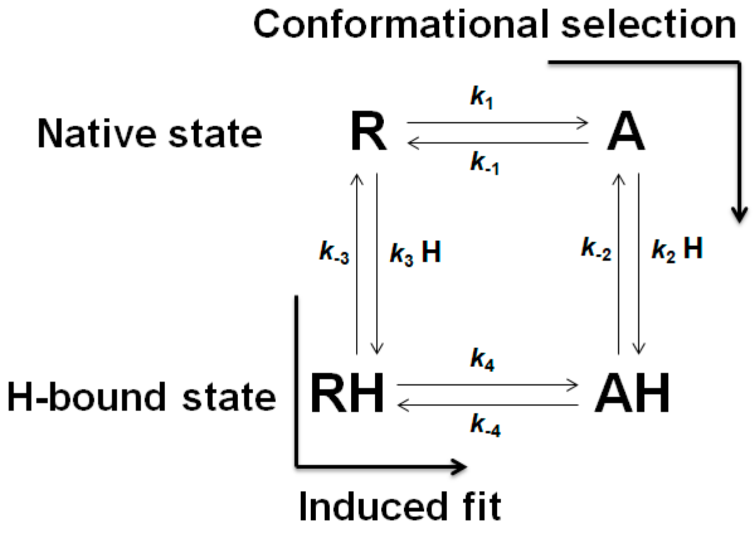

Figure S9. A two-state transition control system regulates antithrombin reactivity. The native equilibrium favors the repressed form (R) by 99.5 % to 0.5 % of the activated form (A). The higher affinity of the A form for heparin (H) drives activation.

S4. Materials and Methods

S4.6. Molecular dynamic simulation of the antithrombin H120V mutant.

1) <u>Minimization script</u>:

```
from openmm.app import *
from openmm import *
from openmm.unit import *

# Load cleaned PDB file
pdb = PDBFile('AT.pdb')
forcefield = ForceField('amber14-all.xml', 'amber14/tip3p.xml')

# Create the system
system = forcefield.createSystem(
    pdb.topology,
    nonbondedMethod=PME,
    nonbondedCutoff=1.0*nanometer,
    constraints=HBonds
)
```

```
# Set up integrator and simulation context
integrator = LangevinIntegrator(310*kelvin, 1/picosecond, 0.002*picoseconds)
simulation = Simulation(pdb.topology, system, integrator)
simulation.context.setPositions(pdb.positions)

# Minimize with verbosity
print("Minimizing energy...")
state = simulation.context.getState(getEnergy=True)
print("Initial energy:", state.getPotentialEnergy())

simulation.minimizeEnergy(maxIterations=10000)

state = simulation.context.getState(getEnergy=True, getPositions=True)
positions = state.getPositions()
print("Final energy:", state.getPotentialEnergy())

# Save minimized structure
with open('AT_WT_final_minimized.pdb', 'w') as f:
    PDBFile.writeFile(pdb.topology, positions, f)
```

2) Molecular Dynamic Simulation script:

```
from openmm.app import *
from openmm import *
from openmm.unit import *
from sys import stdout

# Load the minimized PDB file
pdb = PDBFile('AT_minimized.pdb')
forcefield = ForceField('amber14-all.xml', 'amber14/tip3p.xml')

# Create the system
system = forcefield.createSystem(
    pdb.topology,
    nonbondedMethod=PME,
    nonbondedCutoff=1.0*nanometer,
    constraints=HBonds
)

# Add barostat and integrator
system.addForce(MonteCarloBarostat(1*atmosphere, 310*kelvin))
integrator = LangevinIntegrator(310*kelvin, 1/picosecond, 0.002*picoseconds)

# Create the simulation context
simulation = Simulation(pdb.topology, system, integrator)
simulation.context.setPositions(pdb.positions)
simulation.context.setVelocitiesToTemperature(310*kelvin)

# Total steps: 1000 ns = 500,000,000 steps at 2 fs
n_steps = 500_000_000
save_times_ns = [100, 200, 300, 400, 500, 600, 700, 800, 900, 1000]
```

```
save_steps = [int((ns * 500000) / 1000) for ns in save_times_ns]    # 1000000 steps/ns at 2 fs

# Run the simulation and save structures at each target time
for step in range(1, n_steps + 1):
    simulation.step(1)
    if step in save_steps:
        ns = int(step * 2 * femtoseconds / nanoseconds)
        with open(f'wt_{ns}ns.pdb', 'w') as output:
            PDBFile.writeFile(simulation.topology,
                              simulation.context.getState(getPositions=True).getPositions(),
                              output)
```

3) <u>Fitting two structures using PyMOL to calculate RMSD and visualize heat maps</u>

```
from pymol import cmd

def atomwise_rmsd_heatmap(ref_obj, sim_obj, color_range=(0, 10), show_only_sim=True):
    '''
    Computes per-atom RMSD between ref_obj and sim_obj,
    stores values in B-factor of sim_obj,
    and colors sim_obj as a heatmap.

    Parameters:
        ref_obj (str): name of the reference object
        sim_obj (str): name of the simulated object
        color_range (tuple): (min, max) values for heatmap coloring
        show_only_sim (bool): if True, hides ref_obj and shows only sim_obj
    '''
    coords1 = cmd.get_coords(ref_obj)
    coords2 = cmd.get_coords(sim_obj)
    if coords1 is None or coords2 is None:
        print("Error: Could not get coordinates.")
        return
    if len(coords1) != len(coords2):
        print("Error: Mismatched atom counts.")
        return

    # Compute per-atom displacement
    disps = [(sum((a - b) ** 2 for a, b in zip(p1, p2))) ** 0.5 for p1, p2 in zip(coords1, coords2)]

    # Store in B-factor field of sim_obj
    for i, d in enumerate(disps):
        cmd.alter(f"{sim_obj} and index {i + 1}", f"b={d}")

    print(f"Stored per-atom displacement in B-factor field of '{sim_obj}'.")

    # Reset coloring
    cmd.unset("cartoon_color", sim_obj)

    # Apply spectrum color
    cmd.spectrum("b", "blue_white_red", sim_obj, minimum=color_range[0], maximum=color_range[1])
```

```
    # Visualization
    cmd.hide("everything", "all")
    cmd.show("cartoon", sim_obj)
    if show_only_sim:
        cmd.disable(ref_obj)
```

S4.7 Principal component analysis of WT and H120V simulations.

Python

```
import mdtraj as md
import numpy as np
from sklearn.decomposition import PCA
import matplotlib.pyplot as plt

# Load trajectory and topology
traj = md.load('trajectory.dcd', top='structure.pdb')

# Align to first frame to remove global motion
traj.superpose(traj[0])

# Select Cα atoms
ca_indices = traj.topology.select("name CA")
traj_ca = traj.atom_slice(ca_indices)

# Reshape coordinates: (frames, atoms*3)
X = traj_ca.xyz.reshape(traj_ca.n_frames, -1)

# Mean-center the data
X -= X.mean(axis=0)

# Perform PCA
pca = PCA(n_components=10)
X_pca = pca.fit_transform(X)

# Plot PC1 vs PC2
plt.figure(figsize=(6, 5))
plt.scatter(X_pca[:, 0], X_pca[:, 1], c=np.arange(len(X_pca)), cmap='viridis', s=5)
plt.xlabel('PC1')
plt.ylabel('PC2')
plt.title('PCA Projection (Cα atoms)')
plt.colorbar(label='Frame index')
plt.tight_layout()
plt.savefig('pca_projection.png', dpi=300)
plt.show()

# Plot explained variance
plt.figure(figsize=(6, 4))
plt.plot(np.arange(1, 11), pca.explained_variance_ratio_[:10], marker='o')
plt.xlabel('Principal Component')
plt.ylabel('Explained Variance Ratio')
plt.title('PCA: Explained Variance per PC')
plt.tight_layout()
```

```
plt.savefig('explained_variance.png', dpi=300)
plt.show()
```

4.8 RMSD calculations.

```
from pymol import cmd
import numpy as np

def atomwise_rmsd_heatmap(ref_obj, sim_obj):
    """
    Compute per-residue RMSD between two aligned structures in PyMOL
    and color the reference object by RMSD using a red-white-blue scale.
    Both structures must have the same number of atoms and be aligned.
    """
    # Ensure atom counts match
    if cmd.count_atoms(ref_obj) != cmd.count_atoms(sim_obj):
        print("Error: Mismatched atom counts.")
        return

    # Get atomic coordinates
    ref_coords = cmd.get_coords(ref_obj)
    sim_coords = cmd.get_coords(sim_obj)

    # Compute atomwise RMSD
    diff = ref_coords - sim_coords
    atomwise_rmsd = np.sqrt(np.sum(diff**2, axis=1))

    # Get list of model, chain, resi, and atom names
    model_info = []
    cmd.iterate(f"{ref_obj} and name CA", "model_info.append((model, chain, resi))", space={'model_info':
model_info})

    # Assign RMSD values to B-factors
    for i, b in enumerate(atomwise_rmsd):
        cmd.alter(f"{ref_obj} and id {i+1}", f"b={b}")

    # Color by B-factor using spectrum from blue (low) to red (high)
    cmd.spectrum("b", "blue_white_red", ref_obj, minimum=0, maximum=5)
    cmd.show("cartoon", ref_obj)
    cmd.cartoon("putty", ref_obj)
    cmd.set("cartoon_putty_scale_min", 0)
    cmd.set("cartoon_putty_scale_max", 5)
    cmd.set("cartoon_putty_transform", 1)
    cmd.rebuild()

# Make function accessible in PyMOL
cmd.extend("atomwise_rmsd_heatmap", atomwise_rmsd_heatmap)
```

S4.9. Pearson correlation analysis of residue dynamics.

```
Python
import mdtraj as md
import numpy as np
```

```
import matplotlib.pyplot as plt

# Load trajectories
wt_traj = md.load('WT_trajectory.dcd', top='structure.pdb')
mut_traj = md.load('H120V_trajectory.dcd', top='structure.pdb')

# Focus on last 15% of the trajectory
def crop_last_fraction(traj, fraction=0.15):
    n_frames = traj.n_frames
    start = int((1 - fraction) * n_frames)
    return traj[start:]

wt_traj = crop_last_fraction(wt_traj)
mut_traj = crop_last_fraction(mut_traj)

# Align to first frame to remove global motions
wt_traj.superpose(wt_traj[0])
mut_traj.superpose(mut_traj[0])

# Select only Cα atoms
ca_indices = wt_traj.topology.select("name CA")
wt_ca = wt_traj.atom_slice(ca_indices)
mut_ca = mut_traj.atom_slice(ca_indices)

# Reshape trajectory: frames × (atoms * 3)
wt_xyz = wt_ca.xyz.reshape(wt_ca.n_frames, -1)
mut_xyz = mut_ca.xyz.reshape(mut_ca.n_frames, -1)

# Mean-center
wt_xyz -= wt_xyz.mean(axis=0)
mut_xyz -= mut_xyz.mean(axis=0)

# Compute Pearson correlation matrices
wt_corr = np.corrcoef(wt_xyz.T)
mut_corr = np.corrcoef(mut_xyz.T)

# Average the 3 coordinates per residue
def average_triplet_corr(corr_matrix):
    n_coords = corr_matrix.shape[0]
    n_residues = n_coords // 3
    avg_corr = np.zeros((n_residues, n_residues))
    for i in range(n_residues):
        for j in range(n_residues):
            block = corr_matrix[i*3:(i+1)*3, j*3:(j+1)*3]
            avg_corr[i, j] = block.mean()
    return avg_corr

wt_avg_corr = average_triplet_corr(wt_corr)
mut_avg_corr = average_triplet_corr(mut_corr)

# Compute the delta correlation matrix
delta_corr = mut_avg_corr - wt_avg_corr
```

```
# Save for future plotting
np.save('wt_avg_corr.npy', wt_avg_corr)
np.save('mut_avg_corr.npy', mut_avg_corr)
np.save('delta_corr.npy', delta_corr)

# Optional: visualize delta correlation matrix
plt.figure(figsize=(8, 6))
plt.imshow(delta_corr, cmap='coolwarm', vmin=-1, vmax=1)
plt.colorbar(label='Δ Pearson Correlation (H120V - WT)')
plt.title('Residue-wise Δ Pearson Correlation Matrix')
plt.xlabel('Residue Index')
plt.ylabel('Residue Index')
plt.tight_layout()
plt.savefig('delta_correlation_heatmap.png', dpi=300)
plt.show()
```
